# Pulmonary Delivery of siRNA Anti‐TNFα‐loaded Lipid Nanoparticles for Rapid Recovery in Murine Acute Lung Injury

**DOI:** 10.1002/adhm.202500695

**Published:** 2025-08-07

**Authors:** Qinglin Wang, Jihana Achour, Laila Emam, Younes Louaguenouni, Catherine Cailleau, Françoise Mercier‐Nomé, Séverine Domenichini, Claudine Delomenie, Sezen Gul, Juliette Vergnaud, Nicolas Tsapis, Arnaud Mansart, Djillali Annane, Francois Fay, Elias Fattal

**Affiliations:** ^1^ Université Paris‐Saclay CNRS Institut Galien Paris‐Saclay Orsay 91400 France; ^2^ IHU‐SEPSIS Comprehensive Sepsis Center Raymond Poincaré Hospital (AP‐HP) University of Versailles Saint‐Quentin en Yvelines University Paris Saclay Garches 92380 France; ^3^ General Intensive Care Unit Raymond Poincaré Hospital (AP‐HP) University of Versailles Saint‐Quentin en Yvelines University Paris Saclay Garches 92380 France; ^4^ Université Paris‐Saclay UVSQ INSERM U1173 2I Montigny‐le‐Bretonneux 78180 France; ^5^ UMS‐IPSIT Plateforme MIPSIT Université Paris‐Saclay CNRS Inserm Ingénierie et Plateformes au Service de l'Innovation Thérapeutique Orsay France; ^6^ UMS‐IPSIT Plateforme PHIC Université Paris‐Saclay CNRS Inserm Ingénierie et Plateformes au Service de l'Innovation Thérapeutique Orsay France; ^7^ UMS‐IPSIT Plateforme ACTAGEN Université Paris‐Saclay CNRS Inserm Ingénierie et Plateformes au Service de l'Innovation Thérapeutique Orsay France; ^8^ Institut Universitaire de France (IUF) France

**Keywords:** acute lung injury, lung delivery, nanomedicine, siRNA, TNF‐α

## Abstract

This study investigates the potential of pulmonary delivery of siRNA as an emergency therapy for acute lung injury (ALI). To obtain a quick anti‐inflammatory response, TNF‐α, a critical pro‐inflammatory cytokine, is knocked down for its early involvement in inflammatory responses. Therefore, TNF‐α siRNA‐lipid nanoparticles (LNPs) are designed and characterized for cellular uptake in lipopolysaccharide (LPS)‐activated RAW264.7 murine macrophages, primary alveolar macrophages, and neutrophils from a murine ALI model. Intracellular trafficking and siRNA cytoplasmic release are evaluated in untreated and LPS‐activated RAW264.7 cells. LPS‐activated cells exhibit a fast and strong uptake of LNPs, including in primary cells. In RAW264.7 cells, significant endosomal escape and siRNA cytoplasmic release are observed after 16 h. *In vit*ro efficacy studies reveal consistent TNF‐α inhibition across pre‐, co‐, and post‐incubation protocols, confirming the versatility of siRNA‐LNPs in preventive or curative conditions. After intratracheal administration of TNF‐α siRNA‐LNPs in a murine ALI model, the distribution of LNPs demonstrates an accumulation in immune cells, including macrophages and neutrophils, reducing TNF‐α and IL‐6 levels, indicating a rapid anti‐inflammatory effect. This work underscores the efficacy of TNF‐α siRNA‐LNPs in treating lung inflammatory diseases like ALI and highlights the importance of optimizing LNP distribution and delivery timing to enhance therapeutic outcomes.

## Graphical abstract

In a murine model of acute lung injury, pulmonary administration of lipid nanoparticles carrying TNFα siRNA enables targeted delivery to immune cells. The study emphasises the importance of optimizing LNP distribution and delivery timing to improve therapeutic outcomes and develop emergency therapies for lung hyperinflammation.

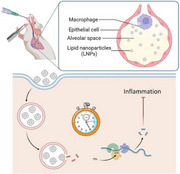

## 1 Introduction

Sepsis is a systemic inflammatory disease caused by severe infections that affects multiple systemic organs, with the lungs being the most common point of entry of the pathogens, making acute lung injury (ALI) patients highly susceptible to developing sepsis^[^ ^1^ ^]^ In ALI, resident and recruited alveolar macrophages are critical in initiating and maintaining pulmonary inflammation.^[^ ^2^ ^]^ Alveolar macrophages from ALI patients have elevated baseline and stimulated secretory levels of pro‐inflammatory proteins, including specific cytokines and chemokines.^[^ ^3^ ^]^ The inflammatory cytokine tumor necrosis factor (TNF‐α) plays a central role in orchestrating the inflammatory immune response,^[^ ^4^ ^]^ allowing the possibility of introducing anti‐TNF‐α therapy, such as monoclonal antibodies, to stop the inflammatory process. Remicade (infliximab) was the first anti‐TNF‐α monoclonal antibody approved for the treatment of rheumatoid arthritis and Crohn's disease^[^ ^5^ ^]^ followed by many other TNF‐α inhibitors, including adalimumab, etanercept, rituximab, and abatacept.^[^ ^6^ ^]^ However, clinical trials based on the systemic administration of anti‐TNF‐α monoclonal antibodies in patients with sepsis‐induced ALI have been unsuccessful in improving survival.^[^ ^7^, ^8^ ^]^ Because of these failures, some investigators have focused on the pulmonary administration of anti‐TNF‐α monoclonal antibodies. Pulmonary administration may be beneficial in the treatment of lung injury. It is a non‐invasive alternative to the systemic route. It offers several other potential advantages, including limited off‐target and off‐organ effects and directly targeting the damaged and inflamed regions of the lung.^[^ ^9^, ^10^ ^]^ However, pulmonary administration of infliximab in vivo in mice showed a minimal reduction in pro‐inflammatory cytokines of only two orders of magnitude.^[^ ^11^ ^]^

Small interfering RNA (siRNA)‐mediated gene silencing offers an alternative therapeutic strategy to overcome inflammatory conditions.^[^ ^12^ ^]^ Several proof‐of‐principle studies have demonstrated the potential of RNA interference to suppress pro‐inflammatory cytokines, particularly TNF‐α.^[^ ^13^, ^14^, ^15^, ^16^, ^17^ ^]^ With this in mind, our laboratory was the first to introduce local pulmonary administration of TNF‐α siRNA delivered by nanocarriers.^[^ ^18^ ^]^ Besides the advantages mentioned above, the pulmonary administration of siRNA reduces interactions with serum proteins that degrade nucleic acids after intravenous administration because the serum is absent on the air side of the lung, and nuclease activity is comparatively low.^[^ ^19^ ^]^ In this first study, we applied PAMAM dendrimers^[^ ^18^ ^]^ and more efficient phosphorus dendrimers to lung delivery of TNF‐α siRNA. These latest dendrimers were functionalized with cationic pyrrolidinium and morpholinium surface groups for better binding to siRNA‐forming dendriplexes.^[^ ^20^ ^]^ These secondary amines have excellent binding with TNF‐α siRNA.^[^ ^21^ ^]^ RAW264.7 cell uptake of pyrrolidinium dendrimers was five and two times higher than that of naked siRNA and morpholinium dendrimers.^[^ ^20^ ^]^ This result may be due to stronger siRNA complexation and improved protection against enzymatic degradation by pyrrolidinium.^[^ ^20^ ^]^ In the LPS‐induced ALI mouse model, nasal instillation of the functionalized dendrimers reduced lung TNF‐α levels by 55% compared to naked siRNA (17%).^[^ ^20^ ^]^ Despite their attractive properties, the knockdown induced by dendriplexes was relatively low.

Lipid nanoparticles (LNPs) have become the gold standard for nucleic acid delivery since the marketing of Patisiran for treating hereditary transthyretin amyloidosis.^[^ ^22^ ^]^ They have been tested for lung administration of mRNA to treat cystic fibrosis.^[^ ^23^ ^]^ However, whether they would be used as an emergency treatment to knock down the strong immune response during lung injury following sepsis is unknown. This work investigates the kinetics of in vitro cytoplasmic release of siRNA from LNPs and subsequent anti‐inflammatory effects. In addition, we examine the cellular uptake in the ALI mouse model and the silencing and anti‐inflammatory effects. We conclude that there is an excellent correlation between in vitro kinetic studies and the early appearance of the first silencing effect in the lungs. These effects are strong at low lipid and siRNA concentrations, opening good perspectives in using the LNP RNA interference approach in emergency treatment of lung inflammation.

## 2 Results and Discussion

### 2.1 Preparation and Characterization of siRNA‐Loaded Nanoparticles

Following Patisiran, the first RNA interference drug as a transthyretin inhibitor, and the mRNA COVID‐19 vaccines,^[^ ^24^ ^]^ most LNP formulations tested preclinically and clinically share a similar composition based on four essential lipids: a structural phospholipid (helper lipid), an ionizable lipid, a PEGylated lipid, and cholesterol. In this study, to formulate LNP, a standard combination, DSPC, DLin‐MC3‐DMA, DMG‐PEG‐2000, and cholesterol was used in a ratio of 9:47:2:42 and an N/P value of 9.6, which closely mirrors the composition of Patisiran.^[^ ^25^ ^]^ This choice was made to extend the scope of our results, particularly those on subcellular release, to other studies using siRNA LNPs targeting macrophages. The formulation and purification methods, including herringbone mixing and centrifugal filtration, were selected to represent the current state‐of‐the‐art techniques in LNP development.

Using those standard formulation processes, we were able to consistently produce batches of LNPs loaded with either TNF‐α or scramble (SCR) siRNAs with hydrodynamic diameters ≈70 nm and low polydispersity, characterizing homogeneous monodisperse populations (**Table** **1**). As expected, those characteristics are similar to siRNA‐loaded LNPs (LNP siRNA) produced by other groups^[^ ^26^ ^]^ and are favorable for cell uptake.^[^ ^27^, ^28^ ^]^ Similarly, zeta‐potential measurements revealed that LNP siRNA presented a characteristic neutral charge at pH 7 and a positive charge ≈30 mV at pH 4 (Table 1) that should facilitate endosomal escape and cytoplasmic release of the siRNAs.^[^ ^25^ ^]^ RNA entrapment efficiencies (EE%) were measured to be over 85% (Figure S1, Supporting Information), aligning with the literature.^[^ ^29^ ^]^ Finally, degradation studies showed that the siRNA molecules encapsulated in LNPs were well‐protected from RNases (Figure S2, Supporting Information).

**Table 1:** Evaluation of size, polydispersity index, zeta potential, and encapsulation efficiency of LNPs.

Sample	Z‐Ave (nm)	PDI	Zeta potential (mV)	EE%
LNP SCR siRNA pH 7	68	0.09	−1.3	95.4
LNP TNF‐α siRNA pH 7	65	0.09	−1.3	88.6
LNP SCR siRNA pH 4	80	0.10	30	/
LNP TNF‐α siRNA pH 4	75	0.12	28	/

### 2.2 Characterizing the In Vitro Anti‐Inflammatory Effect of LNP TNF‐α siRNA

**Figure 1:**
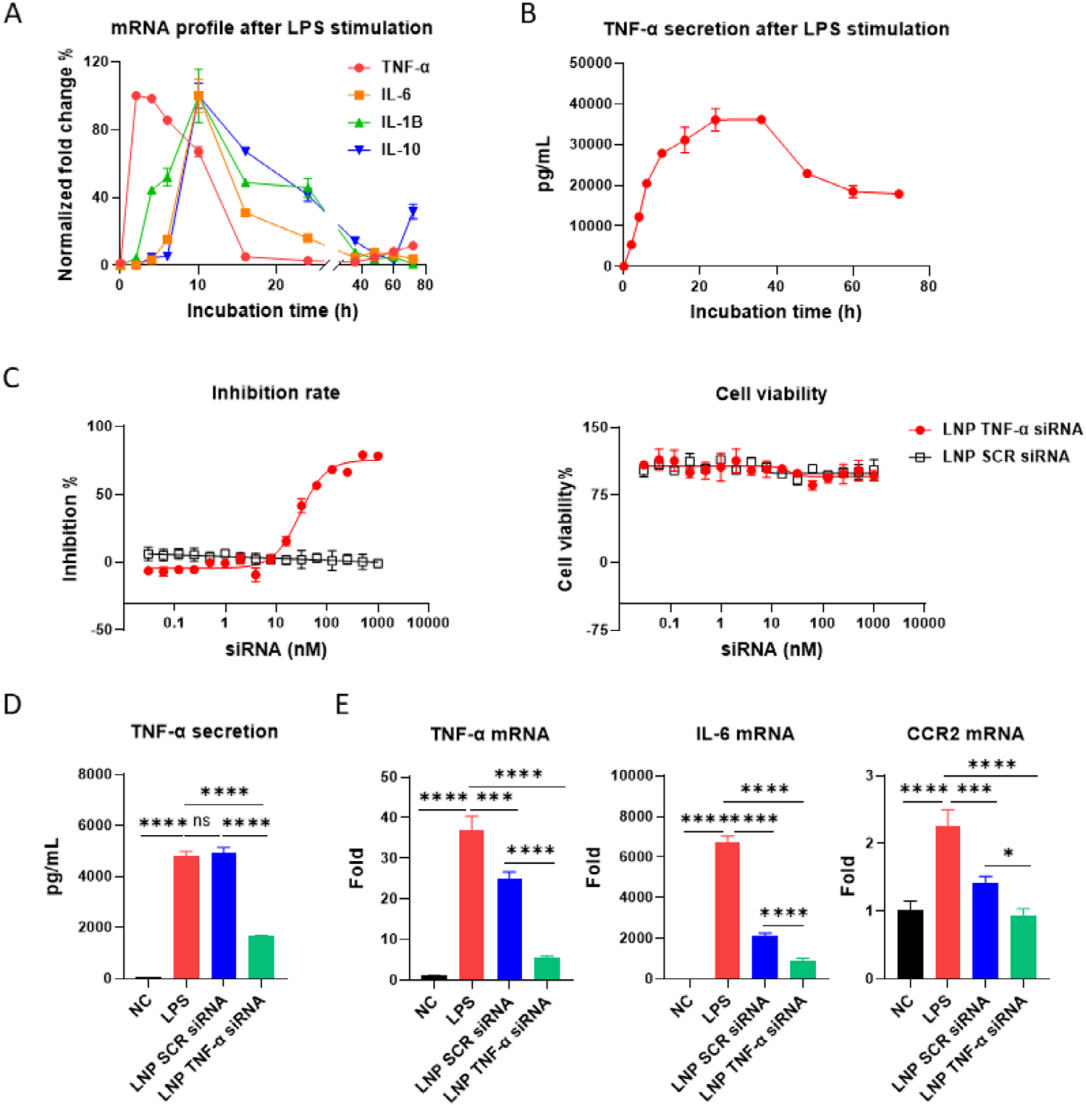
In vitro anti‐inflammatory efficacy of LNP TNF‐α siRNA. A) Normalized transcriptional expression kinetics of TNF‐α, IL‐6, IL‐1β, and IL‐10 in RAW 264.7 cells after LPS stimulation for 2, 4, 6, 10, 16, 24, 36, 48, 60, 72 h (n = 3). B) Secretion of TNF‐α by RAW 264.7 cells after LPS stimulation for 2, 4, 6, 10, 16, 24, 36, 48, 60, 72 h (n = 3). C) Dose‐dependent effect of LNP TNF‐α siRNA on TNF‐α secretion (left) and cell viability (right) on RAW 264.7 cells challenged 6 h of LPS stimulation (n = 3). D) Effect of preincubating LNP TNF‐α siRNA (100 nm) in RAW 264.7 cells on TNF‐α secretion after 6 h of LPS stimulation (n = 3). E) Effect of preincubating LNP TNF‐α siRNA (100 nm) in RAW 264.7 cells on TNF‐α, IL‐6, and CCR2 transcription after 6 h of LPS stimulation (n = 3). Data are displayed as mean ± SD. Statistical significance was assessed via one‐way ANOVA with Tukey's *post hoc* correction *P <0.0332, **P < 0.0021, ***P < 0.00021, **** *P* < 0.0001.

The anti‐inflammatory effect of LNP TNF‐α siRNA was then assessed on RAW 264.7 murine macrophage cell lines incubated with lipopolysaccharide (LPS), known to stimulate Toll‐like receptor 4 (TLR4) present on the cell surface and trigger the activation of several pro‐inflammatory pathways and the production of pro‐inflammatory cytokines. As shown in **Figure** **1**A, upon LPS activation, different cytokines were transcribed sequentially. The mRNA expression analysis showed that TNF‐α transcription peaked ≈80‐fold at 2 h post‐LPS stimulation. In contrast, IL‐6, IL‐1β, and IL‐10 exhibited delayed but substantial peaks at 10 h. Due to its early and rapid expression upon cell activation, TNF‐α is considered a critical mediator of the early stages of the inflammatory response and a suitable therapeutic target.^[^ ^30^ ^]^ Furthermore, results of TNF‐α secretion (Figure 1B) showed an accumulation tendency, with TNF‐α extracellular level peaking at 40000 pg mL^−1^ after 24 h, followed by a plateau and a subsequent decrease. This pattern suggests that TNF‐α is not only a key initiator of inflammation but also maintains its presence in the tissue environment for a prolonged period, reinforcing its relevance as a target for modulating inflammation. Interestingly, while TNF‐α mRNA transcription occurred immediately after LPS exposure, TNF‐α protein secretion lagged. This delay is expected, as the translation and secretion processes naturally require additional time. Those data highlighted the importance of targeting TNF‐α mRNA early in the inflammatory cascade to suppress its transcription and subsequent protein production effectively.

Next, the in vitro efficiency of LNP TNF‐α siRNA was measured in a similar model in which the cells were pretreated 24 h with LNPs before 6 h of LPS stimulation (25 ng mL^−1^). The ELISA results (Figure S3, Supporting Information) demonstrate that LNP‐encapsulated TNF‐α siRNA exerts a net dose‐dependent inhibitory effect on TNF‐α secretion. At lower concentrations (≤ 31.3 nm), TNF‐α levels remain high, like those in the LNP SCR siRNA control group, indicating minimal inhibition. However, as the concentration of LNP TNF‐α siRNA increases beyond 62.5 nm, a marked decline in TNF‐α secretion is observed, with significant suppression occurring at concentrations above 125 nm. These findings suggest that effective siRNA‐mediated knockdown of TNF‐α requires an adequate dosage. In contrast, the LNP SCR siRNA controls show no notable reduction in TNF‐α secretion across all tested concentrations, underscoring the specificity of the TNF‐α siRNA. Curve‐fitting analysis of the dose‐response effect (Figure 1C, left) further confirms that LNP TNF‐α siRNA induces a dose‐dependent inhibition, with an IC_50_ of ≈58 nm. Notably, both LNP formulations exhibited no cytotoxicity, even at high concentrations. Additional viability assays indicated that incubating RAW 264.7 cells with 100 nm of LNPs for up to 72 h did not significantly impact cell viability (Figure 1C, right). Additional enzyme‐linked immunosorbent assay and transcriptional measurements (Figure 1D,E) showed that, although LNP SCR siRNA caused an undesirable in vitro off‐target effect,^[^ ^31^ ^]^ treatment with LNP TNF‐α siRNA not only induced a reduction of TNF‐α secretion and TNF‐α mRNA level but also altered the expression of other pro‐inflammatory cytokines such as IL‐6 and the chemokine receptor CCR2. This suggests that, as expected, by silencing an early‐produced cytokine, our TNF‐α silencing strategy has a broad anti‐inflammatory effect.^[^ ^32^ ^]^ The off‐target effect induced by the scramble siRNA is likely due to 2′O methylation of the antisense strand of the siRNA. Indeed, although 2′O methylation of siRNA is a strategy for designing noninflammatory synthetic siRNA,^[^ ^33^ ^]^ many studies also conclude that this chemical modification provides siRNA immunosuppressor activity,^[^ ^34^ ^]^ which is demonstrated here.

### 2.3 Identifying the In Vitro Kinetics of LNP Cell Uptake, Intracellular Trafficking, and siRNA Cytoplasmic Release

**Figure 2:**
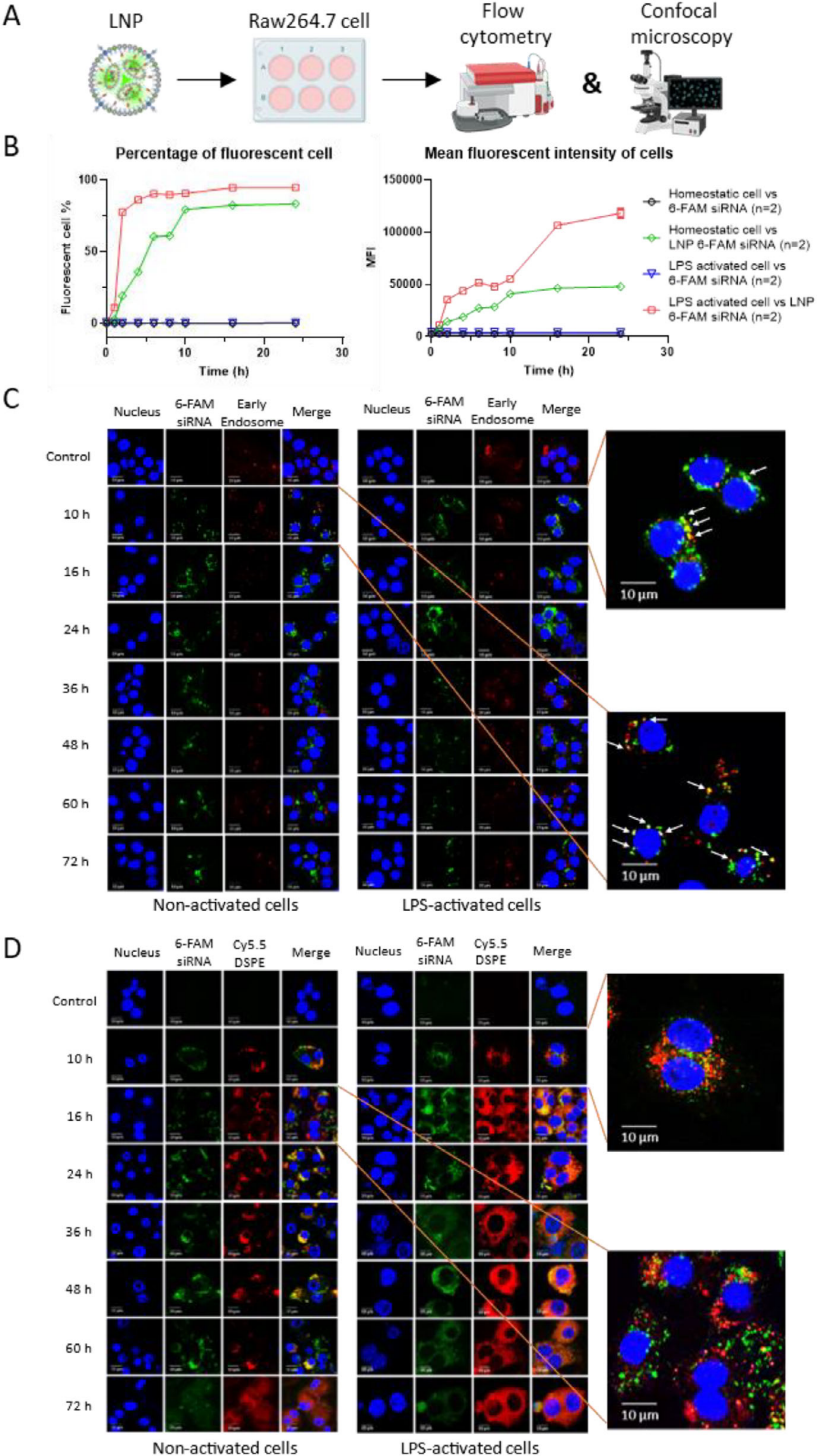
siRNA delivery process by LNPs in RAW 264.7 cells. A) Schematic overview of kinetic experiments. B) Left: The percentage of cell uptake kinetics of fluorescent LNPs in non‐activated and LPS‐activated RAW 264.7 cells (n = 2). Right: Mean fluorescence intensity of RAW 264.7 cells incubated with fluorescent LNP or free siRNA (n = 2). C) Intracellular trafficking by colocalizations of LNPs (labeled by 6‐FAM siRNA, green) and endosomes (red) in non‐activated and LPS‐activated RAW 264.7 cells at different time points (scale bar = 10 µm). D) Intracellular release of siRNA in non‐activated and LPS‐activated RAW 264.7 cells by observing the dissociation of siRNA (labeled by 6‐FAM, green) and LNP (labeled by cy5.5‐DSPE) (scale bar = 10 µm).

Having validated the efficacy of the LNP TNF‐α siRNA platform, we next aimed to assess the kinetics of LNP uptake by macrophages thoroughly. We produced LNPs loaded with 6‐carboxyfluorescein (6‐FAM) labeled siRNA for that purpose. RAW 264.7 cells untreated or pre‐activated with LPS were incubated with fluorescent LNPs or free 6‐FAM‐siRNA (**Figure** **2**A). Flow cytometry was then used to assess the percentage of 6‐FAM‐positive cells at various time points and the cellular mean fluorescence intensity (MFI). As expected, control data showed that incubation with free 6‐FAM‐siRNA or unmarked LNPs did not result in increased fluorescence, whereas incubation with 6‐FAM‐siRNA‐LNPs led to a dose‐dependent cellular fluorescence (Figure S4, Supporting Information). Kinetics measurement (Figure 2B, left) revealed that pro‐inflammatory LPS‐activated macrophages exhibited fast LNP uptake, with almost 80% of the cells being fluorescent after 2 h compared to 20% of the homeostatic cells. However, after 10 h, both phenotypes reached a plateau with ≈80% to 90% of siRNA LNP‐positive cells. The MFI obtained from the same data (Figure 2B, right) revealed that LPS‐activated macrophages consistently showed higher fluorescent values than non‐activated cells, reflecting a faster and more efficient LNP siRNA uptake. Those differences in kinetics between activated and non‐activated cells align with previous results of Khanbeigi et al. with polystyrene nanoparticles.^[^ ^35^ ^]^ Morphological and physiological cellular changes could explain this difference in particle uptake as LPS activation increases cell size and volume, leading to a larger interacting cell surface (Figure S5, Supporting Information). Additionally, activated cells have also been shown to exhibit an increased active endocytosis process compared to non‐activated cells.^[^ ^36^ ^]^ This enhanced endocytic activity in LPS‐activated cells may also be due to increased expression of surface receptors like Toll‐like^[^ ^37^, ^38^ ^]^ and scavenger receptors^[^ ^39^ ^]^ that facilitate the binding and internalization of LNPs. Cell activation is also known to cause cytoskeletal reorganization that forms more endocytic vesicles, such as clathrin‐coated pits,^[^ ^40^ ^]^ and changes in membrane composition that ease vesicle formation.^[^ ^41^ ^]^ Moreover, activated cells have a heightened metabolic state, providing extra ATP for energy‐dependent endocytosis processes, and they upregulate the production of critical endocytic machinery components like clathrin, dynamin, and actin. These combined cellular changes result in a more efficient and robust uptake of LNPs in activated cells compared to their non‐activated counterparts.^[^ ^42^ ^]^ Those in vitro results obtained on RAW 264.7 cells were confirmed on primary alveolar macrophages and neutrophils extracted from LPS‐challenged mice. Data presented in Figure S6 (Supporting Information) revealed that 1h of incubation was enough for the primary alveolar macrophages to take up the siRNA LNPs. Interestingly, unlike RAW 264.7 cells, the MFI value of the primary macrophages presented only a marginal increase (1.17‐fold) within the next 24h following incubation, potentially indicating a saturated uptake. Conversely, neutrophils’ MFI after 1h appeared lower than for macrophages, potentially indicating a minor uptake rate. However, neutrophils’ MFI constantly increased within the experiment (doubling in 24 h), suggesting a more sustained uptake. As lung inflammation is characterized by a massive recruitment of both monocytes and neutrophils, LNP uptake by neutrophils appeared promising.

Due to the specific mechanisms of action and biological fragility of siRNAs, several studies have demonstrated that, more than cell uptake, siRNA intracellular fate, and, in particular, siRNA release from both endo‐lysosomal compartments are critical for successful gene silencing.^[^ ^27^, ^43^ ^]^ Thus, confocal microscopy was subsequently used to characterize 6‐FAM‐labeled siRNA intracellular localization at various time points with different counterstains, such as Cy5.5‐DSPE, RFP‐Rab5a, and RFP‐lamp1 fusion proteins used to stain LNPs, early endosomes, and lysosomes, respectively. Results obtained using RFP‐Rab5a (Figure 2C) show a strong colocalization of siRNA (green) and endosomes (red) signals 10 h after LNPs incubation, with only a marginal account of separated green and red fluorescence. Conversely, images obtained at t = 16 h revealed only a few colocalization features between endosomes and siRNA signals and a more spread‐out siRNA signal, indicating that some siRNA endosomal escape into the cytosol occurred between t = 10 h and t = 16 h. A similar kinetic pattern was observed when studying the colocalization of 6‐FAM‐siRNA with Cy5.5‐DSPE signals (Figure 2D). Following previous results,^[^ ^44^ ^]^ those data indicate that siRNA endosomal escape is concomitant with LNP dissociation, leading to the delivery of free siRNAs within the cytoplasm.^[^ ^45^ ^]^ Importantly, our data also showed that not all siRNAs were released from LNPs, as 6‐FAM and Cy5.5 colocalization patterns were still present in all images taken after that. This observation was confirmed by pictures obtained with RFP‐Lamp1 fusion proteins as counterstain, which revealed patterns of siRNA colocalization with lysosomal markers (Figure S6, Supporting Information). These data indicate that siRNA molecules that do not escape from the endosome are likely to be degraded as the endosomes mature into lysosomes.

Consistent with the findings of Gilleron et al., who demonstrated that LNP internalization in HeLa cells occurs via clathrin‐mediated endocytosis and macropinocytosis, our study observed similar kinetics, though at a slightly slower rate. Gilleron et al. also reported an endosomal escape rate of only 1–2% for siRNA‐gold (2.5 µm) after 6 h of incubation in HeLa cells, with silencing efficiency validated at 72 h.^[^ ^27^ ^]^ In contrast, our study using RAW 264.7 macrophages revealed minimal siRNA release before 10 h but a more pronounced release after 16 h, with a significant amount of siRNA separating from the LNPs. This observation suggests that LNPs have a longer retention time in macrophages, potentially due to the dynamic balance between LNP internalization and exocytosis.^[^ ^46^ ^]^ Furthermore, the more robust and specialized vesicle transport and membrane dynamics in macrophages^[^ ^47^, ^48^, ^49^ ^]^ could enhance later‐stage siRNA release compared to other cell types. These findings imply that early endosomal escape is generally inefficient in macrophages, which could facilitate a more substantial siRNA release at later stages due to differences in endosomal trafficking and maturation.

### 2.4 Identifying the In Vitro Kinetics of LNP siRNA Anti‐Inflammatory Effects

**Figure 3:**
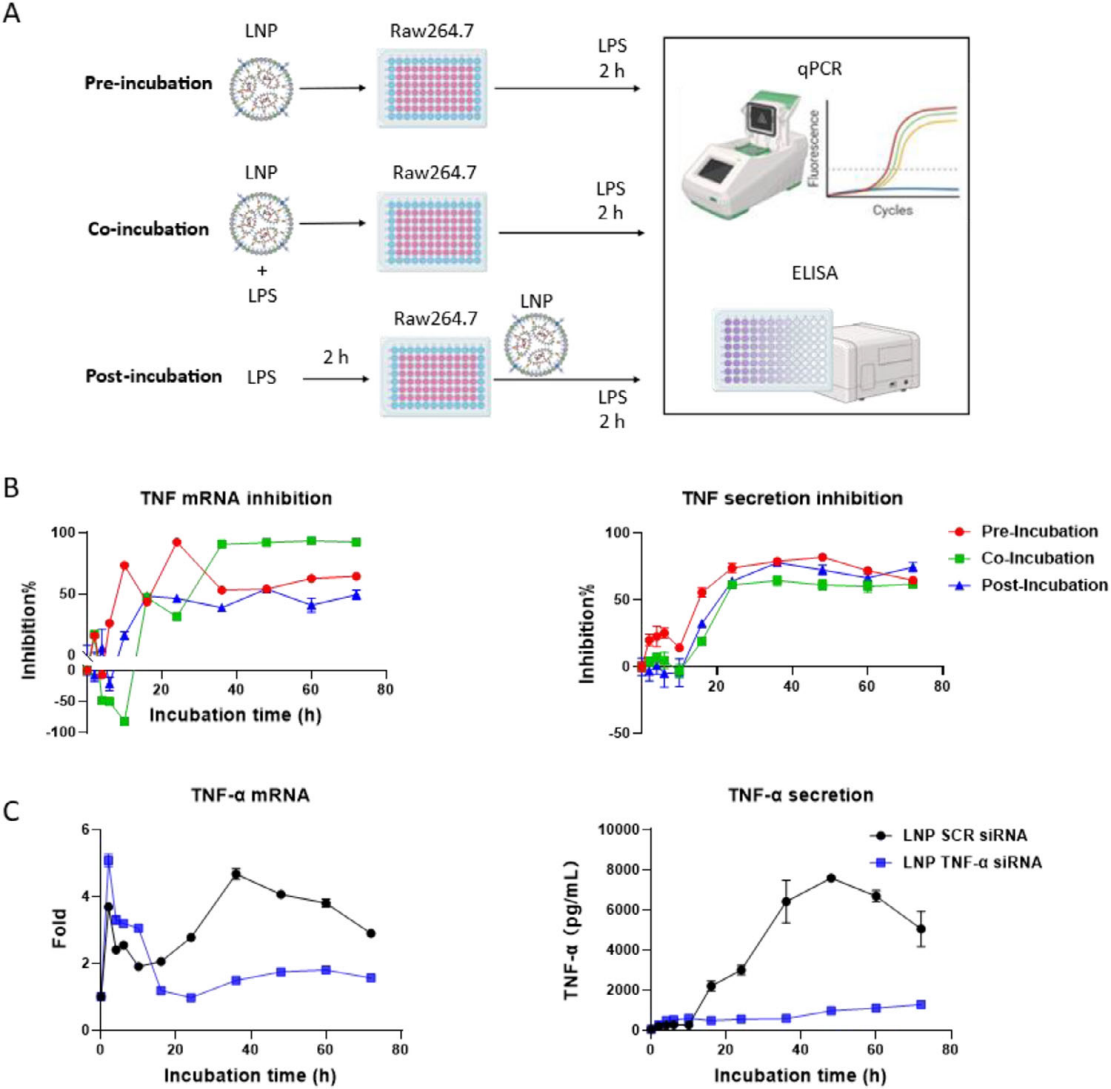
In vitro anti‐inflammatory efficacy of LNP TNF‐α siRNA. A) Schematic illustration of LNP in vitro efficacy evaluation experiments. In pre‐incubation, cells were treated with LNPs for different hours and then activated by LPS for 2 h. In co‐incubation, cells were cotreated with LNPs and LPS simultaneously for different hours and then stimulated by LPS for 2 h. In post‐incubation, cells were first activated for 2 h and treated with LNPs for various hours. Then, cells were stimulated again for 2 h. B) Comparison of TNF‐α transcription and secretion in three treatments (n = 3). C) Comparison of TNF‐α transcription and secretion after incubating LNP TNF‐α siRNA and LNP SCR in inactivated RAW 264.7 cells.

In reviewing prior studies that used siRNA to inhibit the pro‐inflammatory cascade, it was observed that in most experimental protocols, administration was carried out in a prophylactic manner (i.e., before incubation with LPS), which may be helpful for proof‐of‐concept studies but does not accurately reflect clinical scenarios as therapeutic intervention occurs once the inflammation has been initiated.^[^ ^13^, ^20^, ^50^, ^51^, ^52^ ^]^ To address this, we evaluated the in vitro kinetics of LNP TNF‐α siRNA using three treatment protocols: pre‐incubation, co‐incubation, and post‐incubation, as illustrated in **Figure** **3**A. To effectively induce an inflammatory response with minimal impact on cell viability, we activated the cells with a low LPS concentration of 25 ng mL^−1^ in all three protocols. This concentration was chosen to ensure that the observed effects on TNF‐α inhibition were due to the LNP siRNA treatment rather than cytotoxicity from LPS stimulation.

In the pre‐incubation protocol, cells were treated with LNP TNF‐α siRNA for varying incubation periods before being stimulated with LPS for 2 h. This simulates a prophylactic approach, which, while not typical in clinical settings, is commonly used in siRNA studies to evaluate baseline siRNA activity and effectiveness before the onset of inflammation.^[^ ^53^ ^]^ This methodology is frequently observed in siRNA studies aimed at combating inflammatory diseases, as it provides a controlled environment to evaluate the potential of siRNA treatments before the onset of inflammation.^[^ ^51^, ^54^, ^55^, ^56^ ^]^ In the co‐incubation protocol, cells were treated with LNPs and LPS simultaneously for different durations, followed by an additional 2‐h LPS stimulation. This protocol simulates acute inflammatory conditions, where therapeutic and inflammatory stimuli occur concurrently. In the post‐incubation protocol, cells were pre‐activated with LPS for 2 h to induce inflammation, then treated with LNP siRNA for varying durations, followed by a second round of 2 h LPS stimulation. This approach mirrors real‐world clinical scenarios in which treatment is administered after inflammation has already been initiated.

Our results in Figure 3B (details in Figures S7,S8, Supporting Information) revealed that despite the variations in treatment timing, the overall kinetics of the anti‐inflammatory effects of LNP TNF‐α siRNA were remarkably consistent across all protocols. Specifically, TNF‐α transcriptional inhibitions were evident ≈16 h, regardless of the treatment protocol. In comparison, stable inhibitions of TNF‐α protein secretion were observed after 24 h and persisted for the total duration of the 72‐h experimental protocol. As such, the efficacy kinetics also correlate well with the in vitro internalization and intracellular siRNA release data. Our results also highlight the predictable timeline of siRNA‐mediated gene silencing, regardless of the incubation method.^[^ ^27^, ^57^ ^]^

By demonstrating the consistent kinetics of TNF‐α mRNA degradation and protein inhibition across the different treatment protocols, we confirmed that the TNF‐α siRNA‐LNPs were effectively internalized, released from endosomes, and silenced TNF‐α expression promptly. These results also suggest that while the different treatment timings (pre‐, co‐, or post‐incubation) are valuable for studying LNP delivery dynamics, the overall kinetics of siRNA efficacy remain similar.^[^ ^58^ ^]^ This consistency emphasizes the therapeutic potential of using siRNA‐loaded LNPs to suppress inflammation, as they are effective whether administered before, during, or after the onset of an inflammatory event. It also underscores that early intervention, as in pre‐incubation, does not necessarily confer a significant advantage in vitro over post‐incubation regarding outcomes. Finally, these experiments confirm that siRNA‐LNPs effectively inhibit transcription and protein secretion across varying treatment scenarios, making them promising candidates for clinical applications to manage inflammatory diseases.

Our data also revealed that LNPs induced a modest yet significant pro‐inflammatory response in the first hour post‐incubation. To better appreciate this effect, we examined the cytokine expression of unactivated RAW 264.7 cells after LNPs incubation. Data in Figure 3C revealed that incubation with LNP SCR siRNA led to a 5‐fold increase in TNF‐α mRNA levels, leading to significant TNF‐α protein secretion. However, incubating LNP TNF‐α siRNA led to a significantly reduced inflammatory response. TNF‐α protein levels reached only 1300 pg mL^−1^, a ≈6‐fold reduction compared to LNP SCR siRNA. This difference again highlights the protective effect of TNF‐α siRNA, which reduces the production of TNF‐α and mitigates the LNP‐induced inflammatory response.

A detailed analysis of TNF‐α mRNA levels revealed two distinct peaks. An early peak appeared 2 h post‐incubation, and a second peak after 36 h. This biphasic response suggests two distinct pro‐inflammatory mechanisms. Although the immunogenic effects of ionizable lipids^[^ ^59^, ^60^, ^61^ ^]^ or the siRNA^[^ ^62^, ^63^ ^]^ have been documented, the precise kinetics of their influence on cytokine transcription have not been extensively studied. Our data suggest that the first wave of inflammation is likely triggered by the nanoparticles' impact on the cells,^[^ ^64^, ^65^ ^]^ associated with the regulated internalization process.^[^ ^66^, ^67^, ^68^, ^69^ ^]^ In contrast, the second one may be induced by the ionizable lipids, which have been previously reported to generate immune responses, particularly in homeostatic and inflammatory models.^[^ ^60^, ^61^ ^]^ This highlights the need to carefully design and optimize LNP formulations and siRNA design to minimize unwanted immune activation.

### 2.5 Characterizing the In Vivo Anti‐Inflammatory Effect of LNP siRNA in The Acute Lung Injury Model

**Figure 4:**
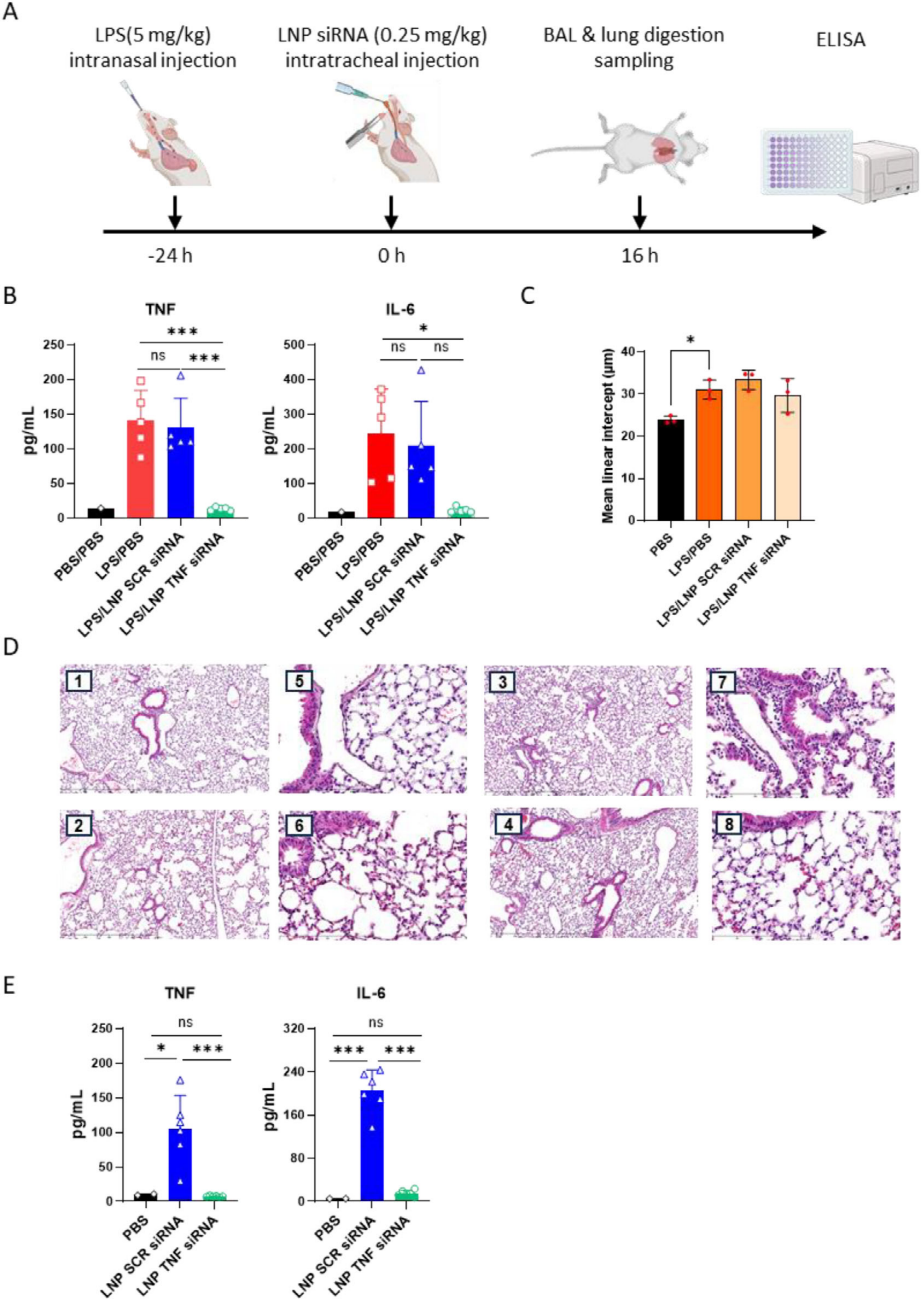
In vivo anti‐inflammatory efficacy in the acute lung injury model. A) Schematic illustration of LNPs in vivo efficacy experiments. B) In vivo efficacy of LNPs on LPS‐induced ALI model (n = 5 for treated mice). C) In vivo effects of in situ pulmonary administration of LNPs on lung morphometric change based on the measurement of the mean linear intercept (n = 3 for treated mice). D) Representative pictures of lung sections fixed, sectioned, and stained with H&E. 1, 5: Tissue from PBS mice showing normal alveolar walls. 2,6: Tissue from the LPS‐induced ALI mouse model, showing damaged alveolar walls. 3, 7, and 4, 8: Tissue from LPS‐induced ALI mouse models, treated 24 h later with SCR siRNA/LNPs or TNF‐a siRNA/LNPs, respectively. 9 Lungs show damaged alveolar walls due to LPS. E) In vivo effects of in situ pulmonary administration of LNPs to healthy mice (n = 5 for treated mice). Data are displayed as mean ± SD. Statistical significance was assessed via one‐way ANOVA with Tukey's post hoc correction *P <0.0332, **P < 0.0021, ***P < 0.00021, **** *P* < 0.0001.

The anti‐inflammatory effect of LNP siRNA was then evaluated in vivo in the LPS‐induced ALI model. Previous studies showed that this model of LPS intranasal administration leads to increased lung secretion of TNF‐α into the bronchoalveolar lavage fluid (BALF) as well as an influx of neutrophils and macrophages with high cellular expressions of pro‐inflammatory markers such as iNOS, CD86, and CD44.^[^ ^70^ ^]^ Importantly, this model does not induce systemic inflammation as no increase of TNF‐α was observed in plasma (Figure S10, Supporting Information). In a proof‐of‐concept experiment, LNP siRNA was administered to the mice in the lungs by intratracheal nebulization, 24 h post‐LPS challenge. Cytokines analysis of the BALF carried out 16 h post‐treatment revealed that the administration of a single dose of LNP TNF‐α siRNA induced a significant anti‐inflammatory effect with the downregulation of the expression and secretion TNF‐α as well as IL‐6 (**Figure** **4**B), demonstrating the exciting perspectives in using the RNA interference approach to treat lung inflammation. These findings were supported by microscopy analysis of BALF content (Figure S11A, Supporting Information). Alveolar macrophage counts data (Figure S11B, Supporting Information) showed an increased number of immune cells in LPS‐challenged mice with LNP TNF‐α siRNA, leading to a reduction (non‐significant) in leukocyte levels compared to the other LPS‐treated groups. Lung histology (Figure 4D) revealed that LPS challenge induced marked alterations in lung architecture, including distorted alveolar spaces, disrupted alveolar walls, and increased inflammatory cell infiltration, particularly around alveolar walls and in interstitial areas, as evidenced by intensified pink/purple staining. Morphometric analysis based on mean linear intercept measurements exhibited no significant differences in alveolar structure across groups after 16 h of treatment (Figure 4C). This suggests that, although LNP TNF‐α siRNA mitigated inflammation, the lung may require additional time to exhibit structural recovery.

As in vitro studies presented above indicated that the LNP siRNA may cause an inflammatory response at the cellular level, a second set of experiments was carried out by administering LNP SCR siRNA and LNP TNF‐α siRNA to healthy mice. Results displayed in Figure 4C revealed that, similarly to in vitro experiments, LNP SCR siRNA administration induced a mild pro‐inflammatory response in the BALF, with elevated secretion of both TNF‐α and IL‐6, averaging 102.5 and 204.5 pg mL^−1^, respectively. Once again, LNP TNF‐α siRNA did not induce a significant pro‐inflammatory reaction, most likely due to the counter‐effect of the TNF‐α siRNA. Importantly, no systemic inflammatory responses were detected in the blood after pulmonary admnistration of LNPs (Figure S10, Supporting Information). Besides, it is important to stress that several studies have been previously conducted on murine ALI using a TNF‐α siRNA; at least two of them could be compared to the present one. In the first one, dendriplexes were produced and administered to mice at a TNF‐α siRNA dose of 2mg/Kg,^[^ ^20^ ^]^ and in the second one, a mannosylated polyplex was designed and administered to the same model at a dose of 0.5mg/Kg.^[^ ^71^ ^]^ In both cases, the maximum inhibition effect was 50%, while in the present case, almost 100% inhibition was obtained with a lower dose of 0.25mg/Kg.

**Figure 5:**
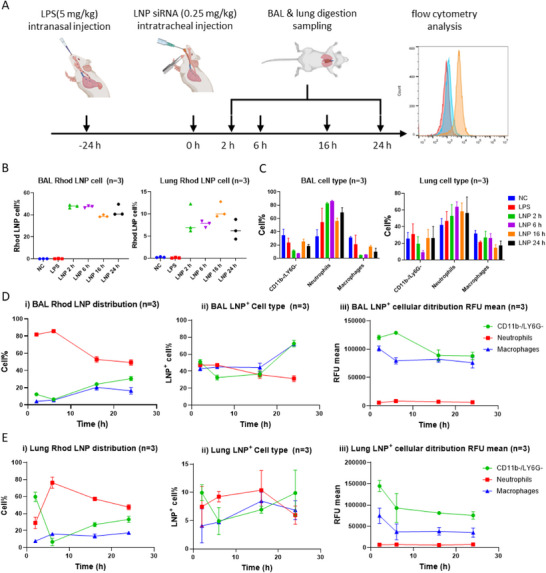
In vivo cellular distribution of LNPs after intratracheal injection. A) Schematic illustration of LNPs in vivo cellular distribution experiments. B) Percentages of LNP‐positive cells in BAL and lung digestion. C) In vivo cellular distribution of LNPs in BAL and lung digestion. D) In vivo cellular distribution of LNPs and temporal changes in BAL. E) In vivo cellular distribution of LNPs and temporal changes in lung digestion. Data are displayed as means ± SD (n = 3).

Having demonstrated the efficacy of LNPs loaded with anti‐inflammatory siRNA to treat lung inflammation, we then chose to characterize the cellular lung distribution of LNP siRNA in both BALF and lung by flow cytometry analyses (**Figure** **5**A). Initial analysis displayed in Figure 5B revealed that after only 2 h, almost 50% of the cells in the BALF had internalized LNPs, indicating a speedy uptake process. Surprisingly, this ratio did not increase with prolonged exposure, most likely indicating that all LNPs had been internalized. Similarly to a previous study on liposomes,^[^ ^70^ ^]^ the ratio of LNP‐positive cells in lung tissue was significantly lower, ≈8%. This difference can be explained by the fact that in lung tissue, only a few layers of cells are directly in contact with the LNPs.

Subsequent analyses were then made using a previously developed gating strategy (Figure S12, Supporting Information) with CD11b^−^/Ly6G^−^ cells defined as homeostatic cells (inactive monocytes/macrophages), CD11b^+^/Ly6G^+^ cells as neutrophils, and CD11b^+^/Ly6G^−^ cells as macrophages.^[^ ^72^, ^73^ ^]^ When analyzing the phenotype of the BALF and lung cells having internalized LNPs (Figure 5Di,Ei), neutrophils appeared to be the main population, accounting for 80% of the LNP‐positive cells after 2 h. Over time, this ratio slightly decreases to ≈50% after 16h. However, when studying the ratio of LNP‐positive cells for each cell population (Figure 5D(ii),E(ii)), it appears that at t = 2 h, every cell population has the same ratio of cells (50%) having internalized LNPs, indicating that our LNPs show an equal affinity for all cell types. As control experiments revealed that LPS administration led to an influx of neutrophils, the high neutrophil percentage displayed in Figure 5D(i),E(i) is most likely an artifact linked to the composition of the cell population. Furthermore, when analyzing the mean fluorescence intensity of LNP‐positive cells, as shown in Figure 5D (iii),E (iii), CD11b^−^/Ly6G^−^ cells and macrophages appear 10 times more fluorescent than neutrophils. This difference can be explained by the difference in internalization mechanisms across cell types, with monocytes and macrophages being more versatile and capable of engulfing a broader range of particles,^[^ ^74^ ^]^ compared to neutrophils.

Together, our data open the exciting possibility of targeting specific cell populations or molecular pathways at different stages of the immune response to improve the efficacy of LNP‐mediated therapies further and provide more robust and precise therapeutic strategies.^[^ ^75^ ^]^ This approach highlights the importance of understanding the cellular distribution and timing of immune responses in LNP‐based therapies and opens avenues for further research in targeting gene expression and modulating immune cell behavior.

## 3 Conclusion

In this study, TNF‐α siRNA was encapsulated in well‐characterized LNPs. The LNPs protected the encapsulated siRNA from degradation and delivered it to the correct site. The cell uptake, trafficking, and release kinetics in homeostatic and LPS‐activated cells were clearly understood. More importantly, the practical time point for LNPs to deliver siRNA to silence the target gene was determined. Although LNPs induced a slight increase in pro‐inflammatory cytokines, this effect was temporary and canceled by the robust silencing effect of TNF‐α. It was emphasized that the LNP TNF‐α siRNA could exert its knockdown effect on TNF‐α in preventive or therapeutic conditions. As a pro‐inflammatory cytokine secreted in the early stages of inflammation, TNF‐α deficiency inhibited the expression of other cytokines. In the murine model of ALI, the proportion of different cell types among LNP+ cells were similar to those in the total live cells. It was shown that LNP TNF‐α siRNA had the same affinity for all cells. Since homeostatic cells and macrophages have strong endocytosis properties, they took up most LNPs. Although neutrophils accounted for most of the total cells and LNP+ cells in the samples, the amount of LNPs internalized by each neutrophil was much lower than that of homeostatic cells and macrophages. Finally, LNP TNF‐α siRNA was highly effective in treating inflammation in the ALI model. In conclusion, our studies demonstrated the timeliness of LNP application in inflammation models and the potential of LNP TNF‐α siRNA as an anti‐inflammatory agent.

## 4 Experimental Section

### Materials

Phospholipids such as 1,2‐distearoyl‐sn‐glycero‐3‐phospho‐ethanolamine‐N‐(Cyanine 5.5) (18:0 Cy5.5 DSPE), 1,2‐distearoyl‐sn‐glycero‐3‐phosphocholine (18:0 PC, DSPC), 1,2‐dioleoyl‐sn‐glycero‐3‐phospho‐ethanolamine‐N‐(lissamine rhodamine B sulfonyl) (ammonium salt) (18:1 Liss Rhod PE), and 1,2‐dimyristoyl‐rac‐glycero‐3‐methoxypolyethylene glycol‐2000, (DMG‐PEG 2000) were purchased from Avanti Polar Lipids. The ionizable lipid D‐Lin‐MC3 was obtained from MedChemExpress. Cholesterol (CHOL) and lipopolysaccharides from *Escherichia coli* O55:B5 (LPS) were provided by Sigma–Aldrich. Collagenase type IV was from Gibco, and DNase I was from Roche. Mouse BD Fc Block, BD Cytofix, and BD CompBeads Anti‐Mouse Ig were purchased from Becton Dickinson. TNF‐alpha Mouse Uncoated ELISA Kit, Fixable Viability Dye eFluor 780, as well as anti‐Ly‐6G (clone 1A8) and anti‐CD11b (clone M1/70) monoclonal antibodies were provided by Thermo Fisher.

All siRNAs used in this study were custom‐synthesized by Eurogentec. The siRNA duplexes consisted of the following sequences. The letter “m” represents 2′‐*O*‐methylation modified ribonucleotide. The siRNA used for confocal microscopy studies (TNF‐α siRNA) was labeled at the 5′‐end of the sense strand with 6‐Carboxyfluorescein (6‐FAM) during the synthesis by the manufacturer.

TNF‐α siRNA:

Sense 5′‐phosphate‐GUCUCAGCCUCUUCUCAUUCCUGdCdT‐3′,

Antisense 5′‐ AGCAGGAAmUGmAGmAAmGAmGGmCUmGAmGAmCmAmU‐3′,

Scramble siRNA:

Sense 5′‐phosphate‐CUUCCUCUCUUUCUCUCCCUUGUdGdA‐3′,

Antisense 5′‐ UCACAAGGmGAmGAmGAmAAmGAmGAmGGmAAmGmUmU‐3′.

### Lipid Nanoparticle Preparation

For each LNP batch, a 1.5 mL ethanol/lipids solution containing the following lipids: D‐Lin‐MC3‐DMA (6.42 mg, 10 µmol), DSPC (1.58 mg, 2 µmol), CHOL (3.47 mg, 9 µmol); and DMG‐PEG 2000 (1.25 mg, 0.5 µmol) was obtained from stock solutions. In parallel, a siRNA aqueous solution was produced by dissolving siRNA (20 nmol, 335 µg) in 4.5 mL sodium acetate (NaAc, 25 mm, pH 4.0), achieving an N/P ratio of 9.6. The lipid solution and siRNA solution were then mixed within a herringbone‐shaped channel micromixer chip (microfluidic ChipShop) using two syringe pumps (New Era NE‐300 InfusionOne) to induce the formation of the lipid nanoparticles. The lipid solution was injected at 1 mL min^−1^, while the aqueous solution was injected at 3 mL min^−1^. The LNP suspension obtained at the end of the microfluidic chip was collected in a 50 mL falcon tube and was immediately diluted with 14 mL of Phosphate‐Buffered Saline (PBS, pH 7.4) to adjust the pH of the solution. The suspension was then filtered using a 0.22 µm polyether sulfone syringe filter (sterile, Millex) to remove any aggregates. Non‐encapsulated siRNAs and free phospholipids were removed by ultracentrifugation using 100 kDa centrifugal filter 50ml tubes (Amicon) that were previously rinsed with 10 mL PBS twice before use. The LNP suspension buffer was exchanged 3 times with PBS by centrifugation‐filtration while discarding the flowthrough. Finally, the LNP suspension was concentrated to a final volume of 2 mL by centrifugation‐filtration and kept at 4 °C until further use.

### Lipid Nanoparticle Characterization

The particle size distribution and polydispersity index (PDI) of the LNPs were determined in PBS using dynamic light scattering, while zeta potential was measured using laser‐Doppler micro‐electrophoresis after a 1‐in‐100 dilution in 1 mm NaCl. All measurements were performed on a Zetasizer Nano ZS (Malvern Instruments).

siRNA encapsulation within the LNP was determined by fluorescent measurement after breaking down the particles. LNPs were first mixed with an equal volume of PBS containing 4% Triton X‐100 to allow lipids and sRNA dissociation. RNA samples were then analyzed using a QuantiFluor RNA System kit (Promega) using the internal siRNA standards in 4% Triton X‐100. Samples and internal standards were mixed with QuantiFluor RNA Dye working solution in black flat‐bottom 96‐well plates and then left to incubate for 5 min at room temperature, protected from light. Each sample's fluorescence (excitation/emission = 490/530 nm) was then measured by a plate reader (PerkinElmer EnVision) using an internal standard curve to quantify RNA concentration. Following those measurements, encapsulation efficiency (EE%) was calculated as follows: EE% = mol of encapsulated siRNA/total siRNA injected × 100%.

The structural integrity of the encapsulated siRNA was measured on agarose gel electrophoresis. 6 µL of each sample, LNP suspension, and free siRNA were first mixed with 4% Triton X‐100 in a volume ratio of 1:1 and left to incubate for 10 min. Samples were then analyzed using gel electrophoresis performed in 3% agarose Tris‐acetate‐EDTA gel containing ethidium bromide buffer and run at 120 V for 50 min. The siRNA bands present in the gel were visualized with ChemiDoc MP Imaging System (Bio‐Rad) and analyzed by Image Lab Software (BioRad) using the electrophoresis DNA 20 bp ladder (JANA Life Sciences) as a marker.

### RNase Degradation Assay of Encapsulated and Free siRNA

To evaluate siRNA stability in LNPs, 500 ng of free siRNA and LNP‐encapsulated siRNA were incubated with RNase A (bovine pancreas, ≥70 Kunitz units/mg protein, Sigma) for 5, 10, 15, 30, 60, and 120 min at 37 °C. RNase was then inactivated by adding 1 µL protector RNase inhibitor (40 U µL^−1^, Roche Life Science). The samples were then assessed by gel electrophoresis.

### In Vitro Model

Murine macrophage cell line RAW 264.7 cells (ATCC TIB‐71) obtained from ATCC (USA) were grown in Dulbecco's Modified Eagle's Medium (DMEM) supplemented with 10% fetal bovine serum, 50 U mL^−1^ penicillin and 50 U mL^−1^ streptomycin at 37 °C with 5% CO_2_. Cells were split twice weekly at a 1/10 ratio using a scraper and used between passages 3 to 14 after thawing.

### In Vitro Model—In Vitro Lipid Nanoparticle Uptake

RAW 264.7 cells were seeded at 2 × 10⁵ cells/well density in 6‐well plates and incubated overnight at 37 °C with 5% CO_2_ to allow the cells to adhere and grow. Cell media were discarded at the beginning of each experiment, and 2 mL of fresh medium containing either LNP loaded with 6‐FAM‐labeled siRNA or free 6‐FAM‐labeled siRNA was added at various concentrations. The cells were then incubated for 2 h before being harvested and washed three times with PBS before flow cytometry analysis (BD Accuri C6). In a parallel experiment, after being seeded, RAW 264.7 cells were first activated with 25 ng mL^−1^ of LPS for 2 h and then let to rest overnight before the beginning of the experiments.

### In Vitro Model—In Vitro Lipid Nanoparticle Cellular Trafficking

LNPs used in this experiment were either labeled by encapsulating 6‐FAM‐labeled siRNA and/or adding a 1% molar ratio of Cy5.5 DSPE in its lipid composition. RAW 264.7 cells were seeded at a 5000 cells/well density in 0.2 mL medium on microscope chamber slides (LAB‐TEK Nunc) and allowed to adhere and grow overnight. One set of cells was non‐activated, while another set was pretreated with 25 ng mL^−1^ LPS for 2 h before resting overnight. Cells were then incubated with diverse fluorescent LNP solutions at a siRNA concentration of 2 µm for 10, 16, 24, 36, 48, 60, or 72 h. For specific samples, 5 µL of CellLight Early‐Endosome‐RFP or CellLight Lysosomes‐RFP (BacMam 2.0, Invitrogen) were added to individual wells according to the manufacturer's instructions. After incubation, the cell media were removed, and the slides were washed twice with PBS. Cells were then fixed within 4% paraformaldehyde solution for 10 min at 37 °C. The slides were then rewashed three times with PBS. Coverslips were then mounted using 10 µL mounting media containing DAPI (ab104139, Abcam) and fixed with nail polish. Microscopy slides were then kept at 4 °C in the dark before being analyzed with an inverted TCS SP8 (gated STED) microscope (Leica, Germany) using a 63×/1.4 HC PL APO CS2 oil immersion objective lens, a 405nm diode laser for DAPI staining (nucleus‐bleu channel) and WLL laser set at 488 nm for 6‐FAM (green channel), 555nm for RFP or 670nm for Cy5.5 (red channels). Fluorescence emissions were collected using a sequential mode with variable beam splitter set respectively between 413‐467 nm, 495‐563/578 nm, 563‐770 nm and 676‐800 nm. 12 bits images were acquired with the Leica SP8 LAS X software (Version 3.5.5; Leica, Germany).

### In Vitro Model—In Vitro Cytotoxicity and Anti‐Inflammatory Effect at the Protein Level

RAW 264.7 cells were seeded into 96‐well culture plates at a density of 1 × 10⁴ cells per well and allowed to adhere and grow overnight. The cells were treated with LNPs and LPS following three different treatment protocols:

Pre‐treatment: Cells were treated with LNPs for different hours, then the culture medium was replaced with fresh medium containing 25 ng mL^−1^ LPS for 2 h. Co‐treatment: Cells were simultaneously treated with LNPs and 25 ng mL^−1^ LPS for different hours. Post‐treatment: Cells were first treated with 25 ng mL^−1^ LPS for 2 h, followed by treatment with LNPs for different hours. Then, cells were stimulated again by 25 ng mL^−1^ LPS.

After each treatment, supernatants were recovered for cytokine secretion measurement by the TNF alpha mouse uncoated ELISA kit (Invitrogen). Cell viability was assessed by incubating them with 200 µL fresh medium and 20 µL of PBS solution containing 5 mg mL^−1^ of 3‐(4,5‐dimethylthiazol‐2‐Yl)‐2,5‐diphenyltetrazolium bromide (MTT). Cells were incubated for one hour at 37 °C to allow the MTT reduction into formazan crystals. At the end of the incubation, the media were withdrawn, and the formazan crystals were dissolved by adding 200 µL of dimethyl sulfoxide to each well. The absorbances of the different samples were measured at a wavelength of 570 nm using a plate reader (Thermo Labsystems Multiskan MS) and compared to untreated cells.

### In Vitro Model—In Vitro Cytotoxicity and Anti‐Inflammatory Effect at mRNA Level

RAW 264.7 cells were seeded with 2 × 10^5^ cells in 6‐well culture plates and a density of 1 × 10⁴ cells per well in 96‐well culture plates and allowed to adhere and grow overnight. The cells were treated with the pre‐treatment, co‐treatment, and post‐treatment regimens described above. At the end of the experiments, RNA was extracted from each well and purified using a NucleoSpin RNA kit (Macherey‑Nagel). The extracted total RNA quality control was measured using a Bioanalyzer 2100 (Agilent) with RNA 6000 Nano Kit (Agilent). Reverse transcription was performed for 1 µg RNA extracts using the iScript cDNA Synthesis Kit (Bio‐Rad). qPCR was then analyzed using a CFX Connect Real‐Time PCR detection system (Bio‐Rad) and Advanced Universal SYBR Green Supermix (Bio‐Rad). The primers used are listed in Table S1 (Supporting Information).

### Primary Cells Model—Neutrophils Isolation

Blood from mice given LPS (5 mg kg^−1^ ‐L2880 Sigma Aldrich) by intranasal instillation was diluted in PBS, layered onto 5 mL of Ficoll‐Paque (Ficoll‐Paque PLUS density gradient, from Cytiva) and centrifuged (400 g, 20 min, RT). Pellets containing mostly neutrophils were collected, washed with PBS (300 g, 5 min, 4 °C), and treated twice with ACK to remove erythrocytes. Cells were then washed twice in RPMI (RPMI 1640 Medium, GlutaMAX Supplement, HEPES 1mM, Sodium pyruvates, and 10% fetal bovine serum (Gibco).

### Primary Cells Model—Alveolar Macrophages Enrichment

In BALF from mice given LPS (5 mg kg^−1^ ‐L2880 Sigma Aldrich) by intranasal instillation, cells were collected after centrifugation (400 g, 5min, 4 °C) and stained 20 min at 4 °C with CD11b microbeads (CD11b MicroBeads, human and mouse, from Miltenyi Biotec) according to manufacturer's instructions. As mouse alveolar macrophages do not express CD11b, the CD11b‐negative fraction was collected after magnetic sorting with the AutoMACS (autoMACS Pro Separator, from Miltenyi Biotec). Cells were washed in PBS (500g, 5 min, 4 °C).

### Primary Cells Model—Ex Vivo Lipid Nanoparticle Uptake

50000 cells/well were seeded in round‐bottom 96 well plates (Well Suspension, from Sarstedt).6‐FAM siRNA LNP uptake in both macrophages and neutrophils was titrated by treating the cells with 2 and 4 µm of 6‐FAM siRNA LNPs, respectively. Neutrophils were incubated with and without LNP encapsulated with 6‐FAM‐labelled siRNA LNPs (4 µm) for 30 min, 1h, 2h, 6h, 12h, or 24h. While macrophages were incubated with 6‐FAM siRNA LNPs (2 µm) for 1, 6, 12, and 24h. Cells were then collected, stained, and analyzed by flow cytometry. Live cells were gated based on the viability dye (Fixable viability Dye efluor 780, Invitrogen), distinguishing live cells from dead cells. Within the live population, neutrophils were identified by gating on Ly6G^+^ cells, and alveolar macrophages were identified as Ly6G‐, CD11b‐, CD11c‐. LNP uptake was assessed by detecting fluorescence from 6‐FAM‐labeled siRNA.

### Animal Experiments

In vivo experimental procedures using C57BL/6 J mice were approved by the CEEA–26 Ethics Committee in Animal Experimentation CAPSUD under the APAFIS#26808‐2020080316289973 v2 protocol. Experiments were conducted following the European guidelines (2010/63/EU) the principles of laboratory animal care, and national French regulations on animal testing (Decree No. 2013–118 of February 1, 2013). Animals were kept under climate‐controlled conditions with a 12 h light/dark cycle, constant temperature (19–22 °C), controlled relative humidity (45–65%), and food and water ad libitum.

### Primary Cells Model—In Vivo Protocols

LPS challenges were carried out by first anesthetizing the mice with isoflurane gas. Once adequate anesthesia was observed, 40 µL of LPS or PBS was introduced intranasally (IN). The dose will be applied with the pipette on the nostrils, drop by drop, carefully to allow the animal to inhale.

LNP administrations were carried out by first anesthetizing the mice by intraperitoneal (IP) injection of a ketamine/xylazine mixture (100 and 10 mg k^−1^g, respectively) and monitored until the disappearance of the plantar reflex. The mice were suspended by their upper teeth with a plastic wire on a support adjusted at a 45–60° angle. The mouth was opened, and the tongue was delicately lifted with blunt forceps and moved to the left. A fiber optic illuminator was then angled toward the neck to light the tracheal orifice. A mouse aerosolizer (Penn‐Century) was then inserted into the trachea. Different LNP suspensions were then aerosolized into the mice's lungs at 0.25 mg kg^−1^ siRNA.

### Primary Cells Model—Cell Sample Collection

At selected time points, animals were euthanized via intraperitoneal (IP) injection of an overdose of pentobarbital (Euthasol 180 mg kg^−1^). After the animal's death was confirmed, the trachea was exposed through an incision and cannulated with a 22‐gauge catheter, and the lungs were flushed twice with 0.7 mL (0.3 mL + 0.4 mL) plus 6 × 0.7 mL cold PBS to recover bronchoalveolar lavage fluid (BALF). The first two lavages were centrifuged at 300 g for 5 min. The supernatants were kept for cytokines quantification by ELISA, while the cellular pellets were pooled with the cells obtained from the subsequent lavages for further analysis.

After BALF collection, the animals were perfused with PBS. Lungs were then collected, diced, and incubated for 40 min at 37 °C in 2 mL of a PBS digestion solution containing collagenase IV (200 µg mL^−1^) and DNase I (10 µg mL^−1^) and let to incubate. The reactions were stopped by adding 4 mL of PBS with 1% FBS and 0.5% BSA. Single‐cell suspensions were then obtained by removing tissue aggregates using cell strainers, and the red blood cells were removed by incubating the cells in sterile water for 30 s before resuspension in PBS.

### Primary Cells Model—Flow Cytometry Analysis

Single‐cell suspensions from BALF and cells were first stained with Fixable Viability Dye eFluor 780 according to the manufacturer's instructions. Cells were then incubated with mouse BD Fc Block for 20 min before being incubated with antibodies targeting CD11b and Ly6G according to the manufacturer's instructions. After incubation, cells were fixed with BD Cytofix fixation buffer for 15 min at 4 °C, washed, and resuspended in FACS buffer. Cell fluorescence was acquired using a BD LSR Fortessa cytometer and analyzed using FlowJo software. A solution containing dead cells, single‐stained compensation beads, and a Fluorescence Minus One cell pool was used as a control to fix the gating processes. The samples were first gated on forward scatter (FSC) versus side scatter (SSC) to select the overall cell population based on size and granularity for flow cytometric analysis. A singlet gate was applied on FSC‐H versus FSC‐A to exclude doublets or cell aggregates. Live cells were gated based on the viability dye, distinguishing live cells from dead cells. Within the population of live cells, CD11b^+^ cells were gated to identify leukocytes. From the CD11b^+^ population, neutrophils were identified by gating on Ly6G^+^ cells. LNP uptake was assessed by detecting fluorescence from Rhodamine. A separate gate was created to differentiate LNP‐positive from LNP‐negative populations.

### Histological Analysis

After treatment and euthanasia, lungs were inflated with 0.7 mL of PBS containing 5 mm EDTA to collect the first bronchoalveolar lavage fluid (BALF1). Two additional lavages were performed and pooled as BALF2. Following transcardiac perfusion with PBS/EDTA, lung tissues were fixed in 4% buffered paraformaldehyde for 24 h, paraffin‐embedded, sectioned (3 µm), and stained with hematoxylin‐eosin (H&E). Slides were scanned using a NanoZoomer S60 Digital Slide Scanner (Hamamatsu, Japan), and images were captured using NDP.view2 software. Lung airspace changes were assessed using the Mean Linear Intercept (Lm) method,^[^ ^76^ ^]^ which estimates average alveolar size. Seven horizontal and vertical lines were overlaid on each image (600×600 µm, ≈ 0.358 mm^2^), and intercepts with alveolar walls were counted. Structures such as bronchioles, blood vessels, and septa were counted only once if partially included. Large airways, major vessels, or damaged alveoli were excluded to avoid bias. At least five regions per mouse were analyzed blindly using ImageJ software (NIH). BAL cells from BALF1 and BALF2 were pelleted by centrifugation (3000 g, 5 min, 4 °C). Supernatant from BALF1 was stored at –20 °C; BALF2 supernatant was discarded. The pellets were combined, red blood cells lysed (5 min), and the remaining cells were washed (400 g, 8 min, 4 °C), resuspended in PBS, and counted using trypan blue and a hemocytometer. A subset of cells (20000–100000) was cytospined onto glass slides (1000 g, RT, 1 min), air‐dried overnight, fixed with 70% ethanol, and stored, protected from dust until H&E staining. Stained slides were scanned and imaged as described above.

### Statistical Analysis

All analyses were done using the GraphPad Prism 6.0 statistical program (San Diego, USA). A *p‐*value *<*0.05 was considered statistically significant (*).

## Conflict of Interest

The authors declare no conflict of interest.

## Supporting information



Supporting Information

## Data Availability

The data that support the findings of this study are available from the corresponding author upon reasonable request.
