# Supplementary material for: Pulmonary Delivery of siRNA Anti‐TNFα‐loaded Lipid Nanoparticles for Rapid Recovery in Murine Acute Lung Injury
Source: Adv Healthc Mater. 2025 Aug 7;14(29):e00695. doi: 10.1002/adhm.202500695 (PMC12616584; doi:10.1002/adhm.202500695)
Supplement: Supplementary file 1 — Supporting Information [file ADHM-14-0-s001.docx]

Pulmonary delivery of siRNA anti-TNFα-loaded lipid nanoparticles for rapid recovery in murine acute lung injury

*Qinglin Wang^a^, Jihana Achour^a,d^, Laila Emam,^b,c,d^, Younes Louaguenouni^a^, Catherine Cailleau^a^, Françoise Mercier-Nomé^e^, Séverine Domenichini^e^, Claudine Delomenie^f^, Sezen Gul^a^, Juliette Vergnaud^a^, Nicolas Tsapis^a,d^, Arnaud Mansart^b,c,d^, Djillali Annane^b,c,d^, Francois Fay^a,d,f^*, and Elias Fattal^a,d^**

^a^Université Paris-Saclay, CNRS, Institut Galien Paris-Saclay, 91400, Orsay, France.

^b^Université Paris-Saclay, UVSQ, INSERM, U1173 2I, 78180 Montigny-le-Bretonneux,

^c^General Intensive Care Unit, Raymond Poincaré Hospital (AP-HP), University of Versailles Saint-Quentin en Yvelines, University Paris Saclay, 92380 Garches, France

^d^IHU-SEPSIS, Comprehensive Sepsis Center, Raymond Poincaré Hospital (AP-HP), University of Versailles Saint-Quentin en Yvelines, University Paris Saclay, 92380 Garches, France

^e^UMS-IPSIT Plateforme MIPSIT, Université Paris-Saclay, CNRS, Inserm, Ingénierie et Plateformes au Service de l'Innovation Thérapeutique, Orsay, France

^f^UMS-IPSIT Plateforme PHIC, Université Paris-Saclay, CNRS, Inserm, Ingénierie et Plateformes au Service de l'Innovation Thérapeutique, Orsay, France

^g^UMS-IPSIT Plateforme ACTAGEN, Université Paris-Saclay, CNRS, Inserm, Ingénierie et Plateformes au Service de l'Innovation Thérapeutique, Orsay, France

^h^Institut Universitaire de France (IUF), France

**Table S1**. Primers used in this study

| TNF-α F | TTGTCTACTCCCAGGTTCTCT |
| --- | --- |
| TNF-α R | GAGGTTGACTTTCTCCTGGTATG |
| IL-6 F | CTTCCATCCAGTTGCCTTCT |
| IL-6 R | CTCCGACTTGTGAAGTGGTATAG |
| IL-1β F | GGTGTGTGACGTTCCCATTA |
| IL-1β R | ATTGAGGTGGAGAGCTTTCAG |
| GAPDH | CATCACTGCCACCCAGAAGACTG |
| GAPDH | ATGCCAGTGAGCTTCCCGTTCAG |
| Mm ACTB F | AGAGGGAAATCGTGCGTGAC |
| Mm ACTB R | CAATAGTGATGACCTGGCCGT |
| 18S F | AGTCCCTGCCCTTTGTACACA |
| 18S R | CGATCCGAGGGCCTCACTA |


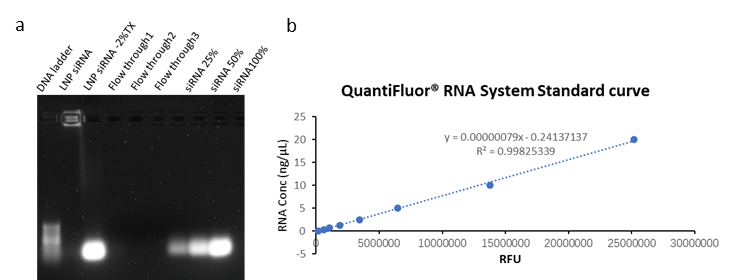


**Figure S1.** LNPs encapsulation efficiency (a). Electrophoresis of free siRNA and encapsulated siRNA. The gel was prepared by dissolving 3 g of agarose powder in 100 mL of Tris-acetate-EDTA (TAE) buffer and stained with ethidium bromide. siRNA-laden lipid nanoparticles (LNPs) and free siRNA (siRNA 25%: 250 ng, siRNA 50%: 500 ng, siRNA 100%: 1000 ng) were mixed with the same volume of 4% Triton X-100 and loaded into the gel. (b). Standard curve for QuantiFluor® RNA System. Fluorescence measurements were obtained using a plate reader with excitation/emission wavelengths of 490/530 nm. The encapsulation efficiency (EE%) of siRNA was calculated based on the quantification of encapsulated RNA relative to total RNA using the formula EE% = (encapsulated siRNA/total siRNA) × 100%.


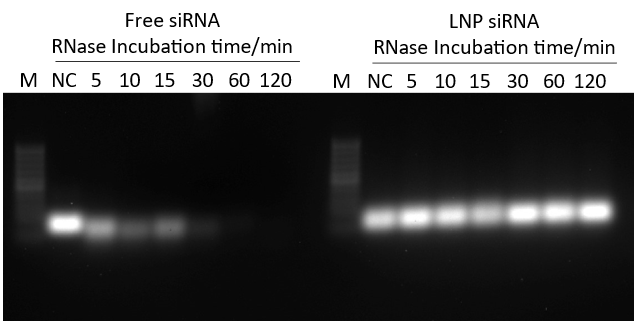


**Figure S2.** Integrity of free and encapsulated siRNAs against RNase A degradations (M: DNA Ladder; NC: negative control, samples without RNase incubation). Free siRNA and LNP-encapsulated siRNA were incubated with RNase A at 37°C for various time points (5, 10, 15, 30, 60, and 120 minutes). Negative controls (without RNase A) were also included. After RNase inactivation and treatment with Triton X-100, the samples were resolved on a 3% agarose gel at 120 V for 50 minutes alongside a DNA 20 bp ladder. The gel was imaged using the ChemiDoc MP Imaging System, and band intensity analysis was performed to assess the degradation of siRNA over time.

**Figure S3.** Dose effect of LNP on anti-inflammatory efficacy. Cells were treated with varying concentrations of LNP (1000, 500, 250, 125, 62.5, 31.3, 15.6, and 7.8 nM) for 24 hours, followed by stimulation with 25 ng/mL LPS for 6 hours. TNF-α secretion was measured by ELISA, and the results show the suppression of TNF-α in response to increasing doses of LNP (n=3).


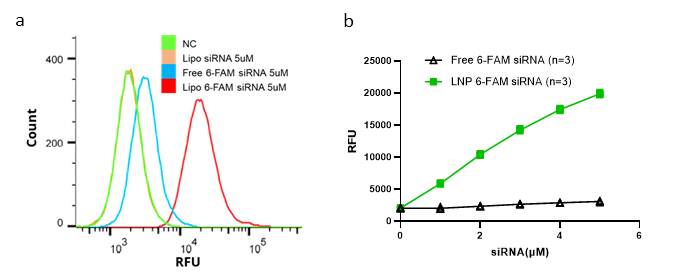


**Figure S4.** LNP cell uptake. Quantifying the siRNA uptake by RAW 264.7 cells using mean fluorescence intensity (MFI). The graph shows a dose-dependent increase in siRNA uptake for both free and encapsulated siRNA, consistently showing higher MFI values, reflecting enhanced cellular uptake.


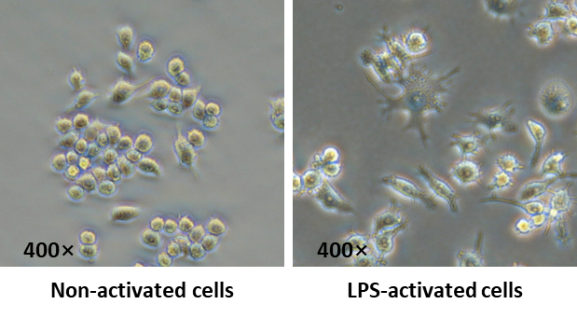


**Figure S5.** Morphology of non-activated (left) and LPS-activated (right) RAW 264.7 cells. RAW 264.7 macrophages were activated with 25 ng/mL LPS for 2 hours, then by refreshing the culture medium and overnight incubation at 37°C with 5% CO₂. The image on the left shows the morphology of non-activated control cells, while the image on the right illustrates the morphological changes of macrophages after LPS activation. Cells were imaged at 400× magnification.


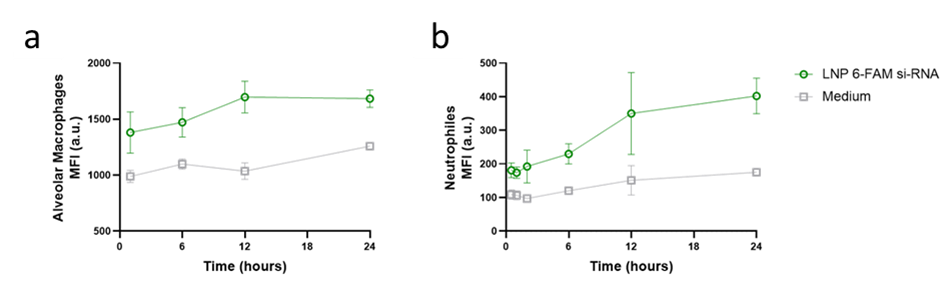


Figure S6. Ex vivo internalization of LNP by primary alveolar macrophages and neutrophils (a) Primary alveolar macrophages extracted from BALF of mice pre-instillated with LPS were incubated with 6-FAM siRNA LNP before flow cytometry analysis (n=3). (b) Primary neutrophils from the blood of mice pre-intillated with LPS were incubated with 6-FAM siRNA LNP before flow cytometry analysis (n=3).


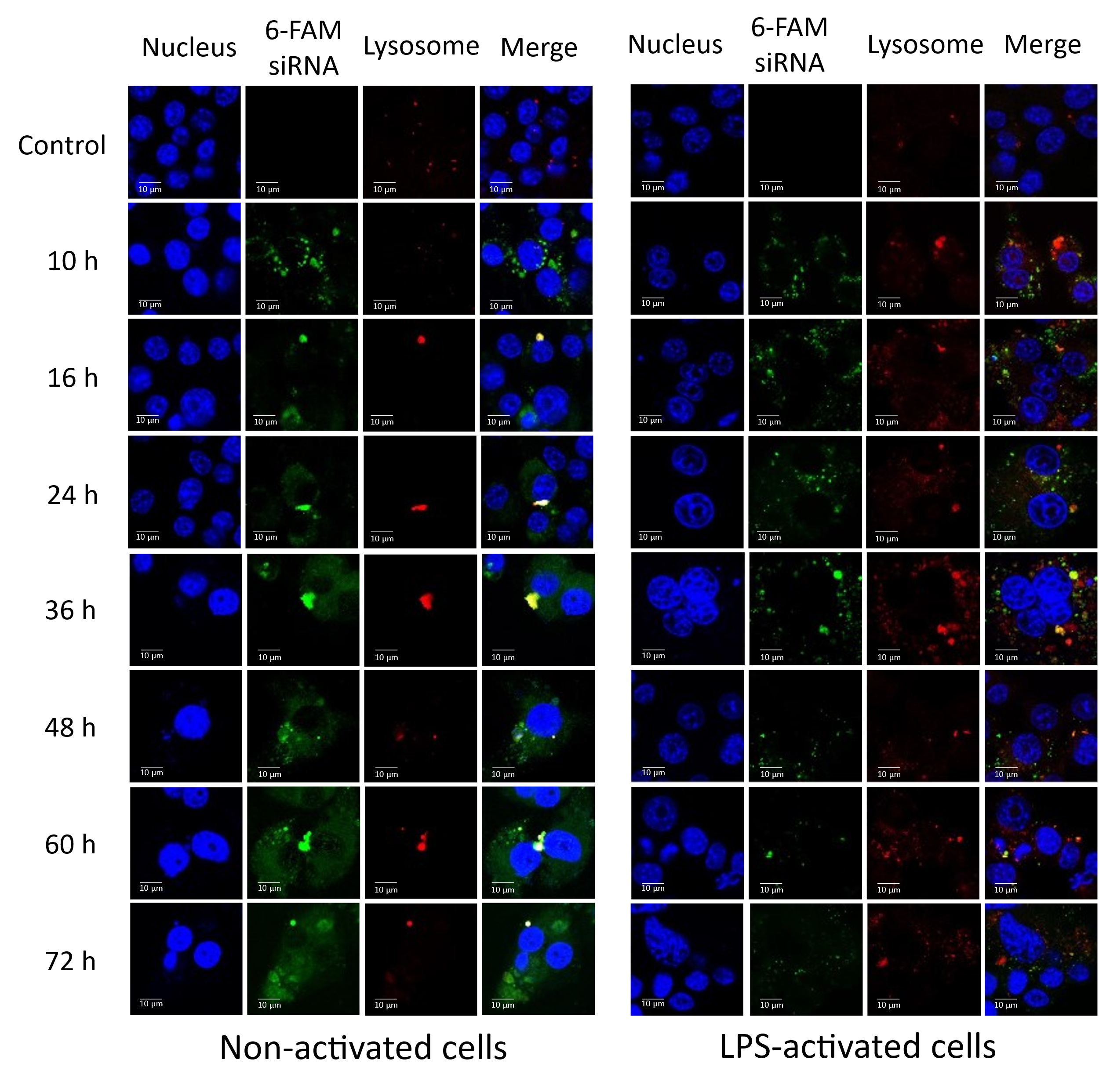


**Figure S7.** The intracellular collocation of LNP and lysosome. RAW 264.7 macrophages were seeded at 5000 cells/well density and treated with LNPs encapsulating siRNA (2 µM). LNPs were labeled with Cy5.5 DSPE, and siRNA was conjugated with 6-FAM for fluorescence tracking. One set of cells on the left was non-activated, while another set was pre-treated with 25 ng/mL lipopolysaccharide (LPS) for 2 h before LNP treatment. After incubation with LNPs for 10, 16, 24, 36, 48, 60, and 72 hours, cells were fixed, stained with DAPI, and prepared for microscopy. Lysosomes were labeled with CellLight™ Lysosomes-RFP (5 µL/well) 24 hours before fixation. Colocalization was assessed using confocal microscopy (Leica TCS SP8). The images show 6-FAM-labeled siRNA (green) and lysosomes (RFP, red). DAPI staining (blue) highlights cell nuclei.


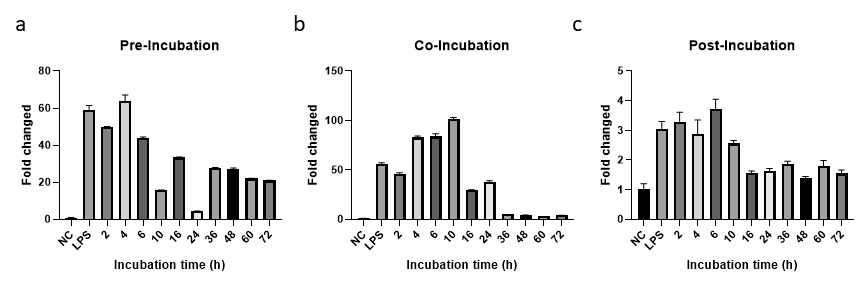


**Figure S8.** Effects of LNP treatment on TNF-α mRNA under different treatment protocols (pre-treatment, co-treatment, and post-treatment) in RAW 264.7 macrophages. Cells were seeded at 1 × 10⁴ cells per well in a 96-well plate and subjected to the following treatments: (a) Pre-treatment with LNPs for various time points before stimulation with 25 ng/mL LPS for 2 hours (n=3); (b) Co-treatment where cells were exposed to both LNPs and 25 ng/mL LPS simultaneously for different durations (n=3); and (c) Post-treatment where cells were first stimulated with 25 ng/mL LPS for 2 hours, followed by treatment with LNPs for various durations. After treatments, cells were collected to measure TNF-α mRNA (n=3).


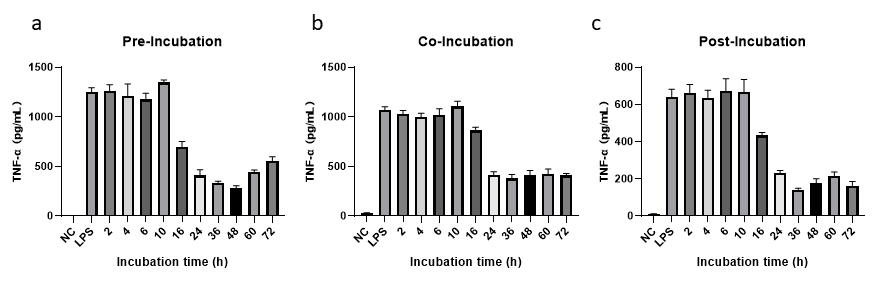


**Figure S9**. Effects of LNP treatment on TNF-α secretion under different treatment protocols (pre-treatment, co-treatment, and post-treatment) in RAW 264.7 macrophages. Cells were seeded at 1 × 10⁴ cells per well in a 96-well plate and subjected to the following treatments: (a) Pre-treatment with LNPs for various time points before stimulation with 25 ng/mL LPS for 2 hours (n=3); (b) Co-treatment where cells were exposed to both LNPs and 25 ng/mL LPS simultaneously for different durations (n=3); and (c) Post-treatment where cells were first stimulated with 25 ng/mL LPS for 2 hours, followed by treatment with LNPs for various durations. After treatments, supernatants were collected to measure TNF-α secretion level (n=3).

**Figure S10**. Plasma level of TNFα in LPS-induced ALI mouse model by intranasal administration. TNFa level in plasama were mesured by ELISA 16h after treatment (n=3).


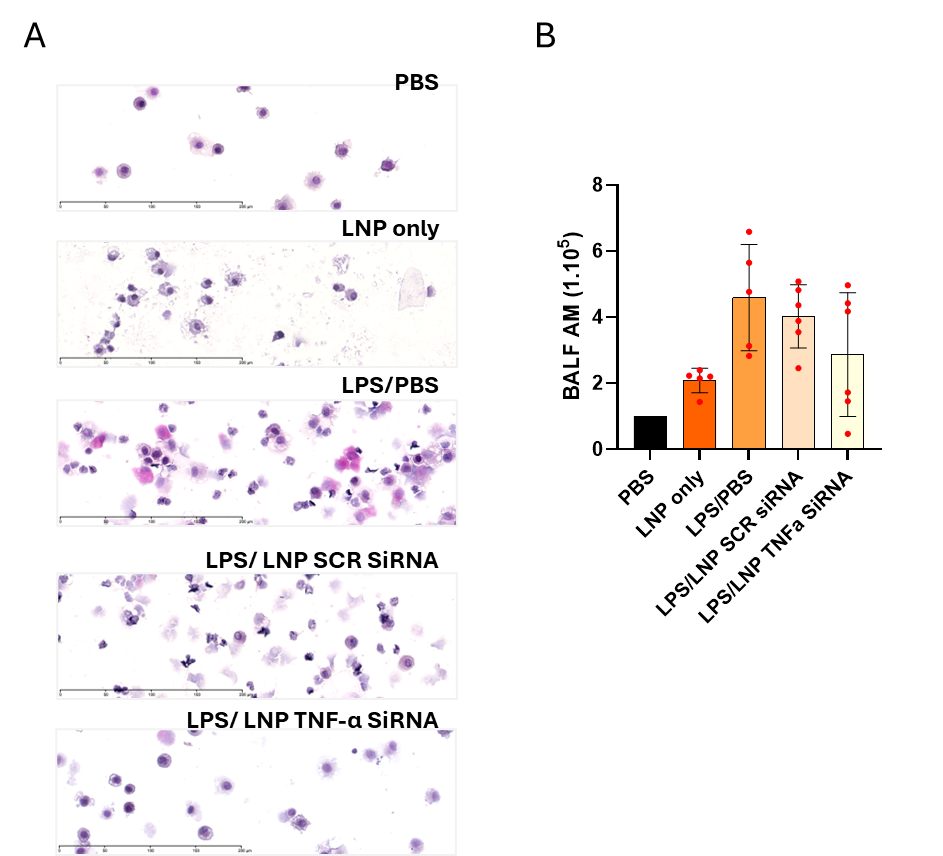


**Figure S11.** Macrophage count in BALF. (A) Representative pictures of cells from BALF collected 16 hours post-instillation and stained with H&E. (B) Changes in the numbers of macrophages in BALF mice.


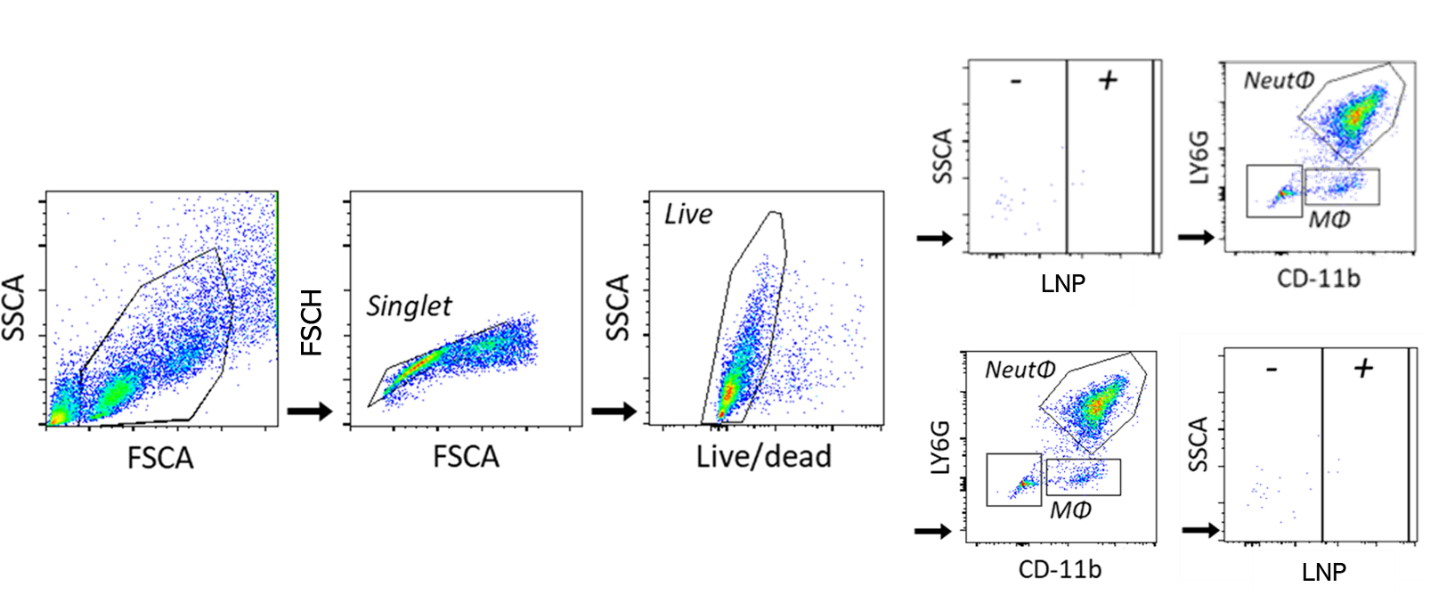


**Figure S12**. Flow cytometry gating strategy for the identification of cell populations and LNP uptake. Cells were first gated based on forward scatter (FSC) and side scatter (SSC) to define the overall population based on size and granularity. Singlets were gated to exclude doublets using FSC-H vs FSC-A. Live cells were gated using a viability dye to distinguish viable from non-viable cells. Monocytes were identified by CD11b expression, and within the CD11b+ population, neutrophils were distinguished from monocytes/macrophages using Ly6G and CD11b markers. LNP uptake was assessed by measuring liposome fluorescence, and a gate was applied to quantify the percentage of neutrophils and macrophages that had taken up LNPs.
